# Comprehensive Analysis Based on the Cancer Immunotherapy and Immune Activation of Gastric Cancer Patients

**DOI:** 10.1155/2023/4674536

**Published:** 2023-03-06

**Authors:** Feng Jiang, Qilong Ma

**Affiliations:** ^1^Department of Oncology, Zhongda Hospital, Southeast University, Nanjing 210009, China; ^2^Department of General Surgery, Jining Hospital, Nanjing University, Nanjing, Jiangsu, China

## Abstract

When it comes to aggressiveness and prognosis, immune cells play an important role in the microenvironment of gastric cancer (GC). Currently, there is no well-established evidence that immune status typing is reliable as a prognostic tool for gastric cancer. This study aimed to develop a genetic signature based on immune status typing for the stratification of gastric cancer risk. TCGA data were used for gene expression and clinical characteristics analysis. A ssGSEA algorithm was applied to type the gastric cancer cohorts. A multivariate and univariate Cox regression and a lasso regression were conducted to determine which genes are associated with gastric cancer prognosis. Finally, we were able to produce a 6-gene prognostic prediction model using immune-related genes. Further analysis revealed that the prognostic prediction model is closely related to the prognosis of patients with GC. Nomograms incorporating genetic signatures and risk factors produced better calibration results. The relationship between the risk score and gastric cancer T stage was also significantly correlated with multiple immune markers related to specific immune cell subsets. According to these results, patients' outcomes and tumor immune cell infiltration correlate with risk scores. In addition, immune cellular-based genetic signatures can contribute to improved risk stratification for gastric cancer. Clinical decisions regarding immunotherapy and followup can be guided by these features.

## 1. Introduction

The World Health Organization estimates that there are approximately 1,089,103 new cases of gastric cancer (GC) every year, ranking it sixth among newly diagnosed cancers [1]. Each year, approximately 769,000 people die from GC, making it the third most common type of cancer [2]. In Asia, Eastern Europe, and South America, gastric cancer is endemic, with a wide range of incidences worldwide [3]. Gastric cancer pathogenesis involves a number of factors, such as Helicobacter pylori, atrophic gastritis, intestinal metaplasia, and dysplastic tissues of the gastrointestinal tract [4]. Growth pattern, differentiation, and molecular pathogenesis differ significantly between stomach cancers. Adenocarcinomas account for more than 90% of stomach cancers [5].

Mutant cells are normally recognized and eliminated by immune cells, preventing cancer development. In many cases, cancer cells are unable to be detected by the immune system, which allows them to reproduce rapidly. PD-L1 occurs when cytotoxic T lymphocytes (CTL) encounter cancer cells and secrete interferon (IFN)-c and activate the JAK-STAT pathway. The PD-L1 receptor blocks inhibitory signals, which prevents T cells from killing tumor cells [6]. In many clinical studies, immunotherapy targeting PD-L1 has been shown to be effective [7]. Next-generation sequencing (NGS) has revealed a growing number of immune checkpoint molecules, including PD-L2, CTLA-4, CD80, and PD-L1 [8].

On a global scale, genome analysis is the most popular method for determining new biological targets for GC patients. There have been numerous studies examining the interactions between cancer cells and their microenvironment. There have been several immunotherapeutic treatments proposed for the treatment of GC since the immune system regulates the progression of the disease [9]. Many types of cancer, including advanced gastric cancer, respond strongly and durably to antibodies that block the PD-1/PD-L1 pathway. Immune checkpoint inhibitors are not effective biomarkers for predicting the efficacy of immune checkpoint inhibitors based on anti-PD-1, anti-PD-L1, and anti-MSI status, as well as a mutational burden [10]. In order to maximize the therapeutic benefits of checkpoint immunotherapy, predictive biomarkers are important. TME may play an important role in checkpoint inhibitor immunotherapy, according to emerging evidence [11]. To identify novel immunotherapeutic targets for GC, it is necessary to characterize the immunophenotypic characteristics of GC and better understand immune cells' regulatory functions [12]. This study examined the relationship between GC and immunity by dividing the GC cohort into different subtypes. To improve our understanding of the genetic factors involved in the occurrence and development of GC, we constructed a prognostic prediction model.

## 2. Materials and Methods

### 2.1. Dataset Downloaded

Data on mRNA expression and clinical information on GC patients were downloaded from the TCGA database, which contains 375 GC patients. TCGA and GTEx datasets, which include a total of 391 normal stomach tissues, were used for the analysis of expression data and clinical characteristics of normal stomach tissues. In addition, the immune-related genes were obtained from the online database.

### 2.2. Differential Expressed Analysis Based on the GC Cohort in the TCGA Database

A different expressed analysis was conducted between the TCGA GC cohort and the normal cohort using TCGA and GTEx database in R using the mana expression data obtained from the TCGA database. *P* value < 0.05 was considered as static significance.

### 2.3. Pathway Function Analysis Based on Key Genes

Annotating key genes and exploring candidate gene functions in R was carried out using the “ClusterProfiler” package. We then identified related functional categories by using the Kyoto encyclopedia of genes and genomes (KEGGs) and gene ontology (GO). *P* values and *q*-values less than 0.05 were considered statistically significant for GO-enriched pathways and KEGG-enriched pathways. Based on the gene sets, the gene set enrichment analysis (GSEA) identified GO terms and KEGG pathways. The 50 best terms from each subtype were selected based on significance. Gene set variation analysis (GSVA) was used to measure gene set enrichment. It is possible to determine the biological function of a sample by converting gene-level changes into pathway-level changes through comprehensive scoring of gene sets of interest. A comprehensive assessment of potential biological functional changes was performed for various samples using the GSVA algorithm.

### 2.4. The Classification of Immune Subtypes in the GC Cohort

In ssGSEA, overexpression measures are calculated based on all other genes within the genome using a rank-based approach. The next step in the process was to classify GC using 29 immunobiosignature enrichment levels (ssGSEA scores) and determine tumor purity and immune score for each GC.

### 2.5. A Prognostic Prediction Model Based on Genes Related to GC and Immune Function

For the purpose of exploring the genes that are closely related to gastric cancer and immune, an analysis of univariate and multivariate Cox regressions as well as lasso regressions was conducted to identify genes closely associated with gastric cancer prognosis. Each patient was assigned a risk score in the immune-related genes.

### 2.6. Immune Cell Infiltration

From RNA-seq data from different subgroups of GC patients, the relative proportions of 22 immune-infiltrating cells were calculated using the CIBERSORT algorithm. Based on the results of Spearman's correlation analysis, we determined that there was a significant relationship between gene expression and immune cell infiltration.

### 2.7. Drug Sensitivity Analysis

Based on the drug sensitivity genomics in the cancer database, the “pRRophetic” R package (GDSC) was used to predict chemosensitivity for each tumor sample. Regression analysis was performed to determine each drug's IC50 value. The accuracy of regression and prediction was tested ten times using GDSC training data. A default value was set for all parameters, including the “battle” parameter, which averaged gene expression across replicates to eliminate batch effects.

### 2.8. Statistical Analysis

Using log-rank tests and Cox proportional hazards models, survival curves were calculated and compared in a multivariate analysis. Statistical significance was defined as a *P* value of less than 0.05 on both sides of the test. All analyses were conducted using R software.

## 3. Results

### 3.1. Immune Subtype Analysis Showed the Two Immune Subtypes of GC Cohort

**Figure 1:**
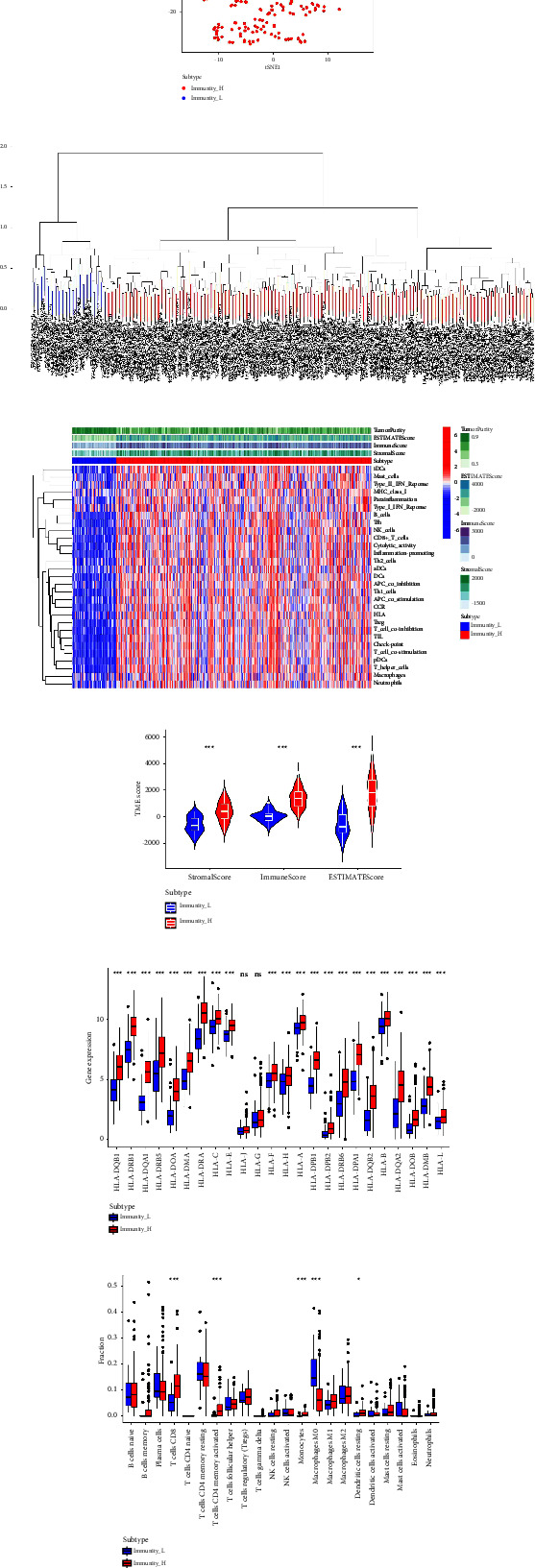
(a) The PCA analysis reveals the different immune subtypes of GC cohort; (b) the different immune subtypes were divided by ssGSEA algorithm; (c) the different immune cell infiltration between Immunity_L and Immunity_H subtype; (d) the stromal score, immune score, and estimate score between Immunity_L and Immunity_H subtype; (e) the expression level of HLA encoding genes between Immunity_L and Immunity_H subtype; (f) the immune cells analysis between Immunity_L and Immunity_H subtype.

Our first step was to examine 29 immuno-related genomes, each representing a different type, cascade and function of immune cells. In the TCGA dataset containing GC samples, immune cell expression profiles were analyzed using ssGSEA. Based on the expression level of immune cells, GC cohorts were divided into high immunity (Immunity_H) and low immunity (immune_L) groups (Figures 1(a) and 1(b)). Heat maps showed that Immunity_H subtypes were associated with highly infiltrated immune cells and active immune pathways. Moreover, Immunity_L subtypes are linked to low immune cell infiltration, an indication of an immune cold. In addition, it was discovered that patients with renal cell carcinoma had different immune components based on their immune subtype. It is evident from the heatmap that the immunological subtype Immunity_L is more enriched in tumor purity than the immunological subtype Immunity_H (Figure 1(c)). Compared with Immunity_L, the Immunity_H subtype had higher scores on stromal, immune, and estimated components (Figure 1(d)). It is essential for immunotherapy targeting immune checkpoints that tumor-associated antigens are presented by MHC class I complexes during immune surveillance. As a result, we measured the levels of 24 human leukocyte antigens (HLAs). Immunity_L had reduced expression levels of most immune HLA genes, suggesting that tumor cells are evading antigen presentation by impaired antigen presentation (Figure 1(e)). In addition, the immune cells distribution analysis revealed that CD8^+^ T cells, CD4^+^ T cells, monocytes, DC cells, and M0 macrophages showed significantly different between Immunity_L and Immunity_H groups (Figure 1(f)).

### 3.2. Immunity_L and Immunity_H Subtypes Differ in a Large Number of Immune-Related Genes

**Figure 2:**
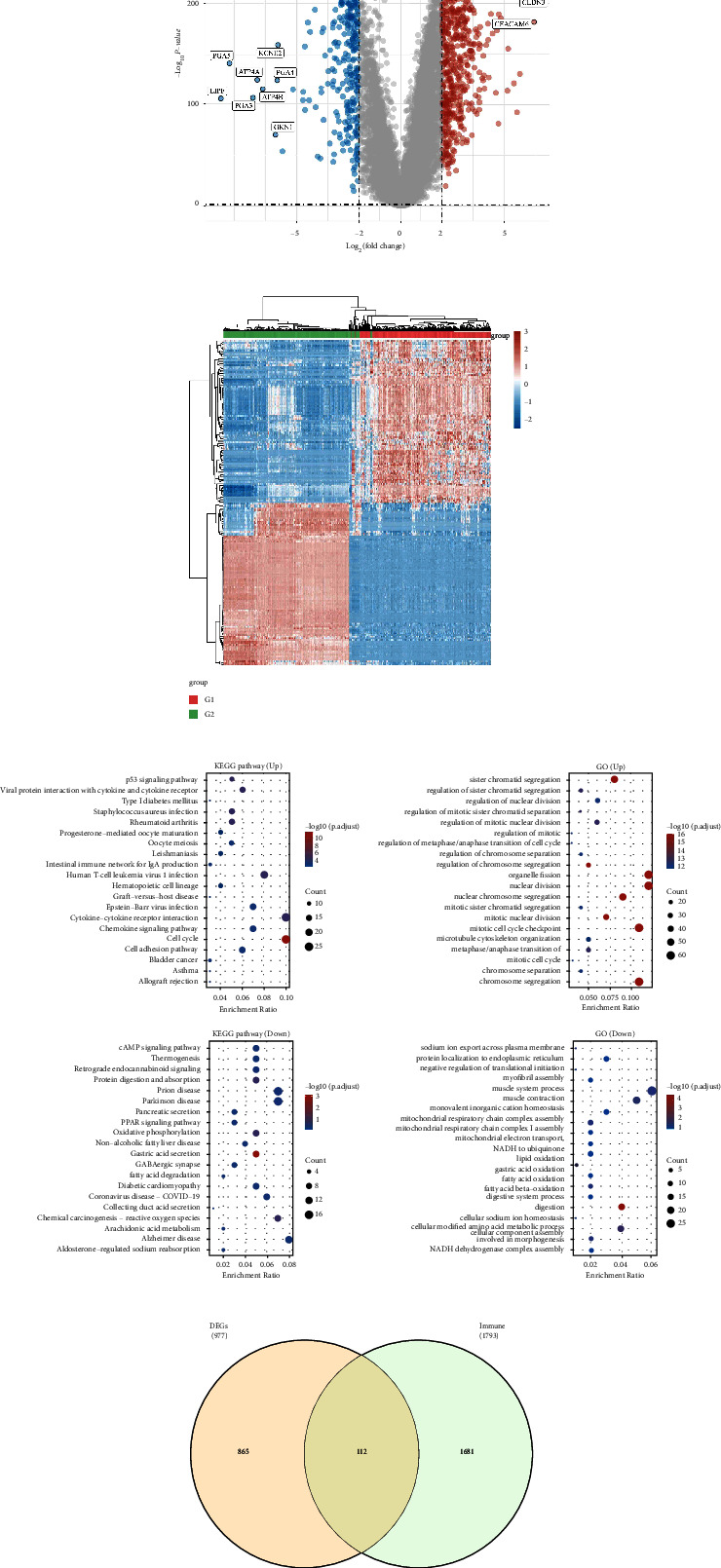
(a) The differential expressed analysis between Immunity_L and Immunity_H subtype; (b) the heat map demonstrated the gene expression level between Immunity_L and Immunity_H subtype; (c) the GO and KEGG enrichment analysis based on the differential expressed genes; (d) the Venn diagram demonstrates the genes involved in immune-related genes and differential expressed genes.

By performing a differential expression analysis, we were able to identify the genes that are important to both Immunity_L and Immunity_H subtypes. Based on the results, 977 genes were identified as differential genes, including 546 up-regulated genes and 431 down-regulation genes (Figures 2(a) and 2(b)). Our next step was to perform GO and KEGG enrichment analyses to identify pathways that play a key role between subtypes. The results demonstrated that up-regulated genes are closely associated with sister chromatid segregation, regulation of sister chromatid segregation, p53 signaling pathway, regulation of nuclear division, and progesterone-mediated oocyte maturation. In addition, the results revealed that down-regulated genes are closely associated with the camp signaling pathway, protein digestion and absorption, negative regulation of translational initiation, muscle system process, myofibril assembly, and fatty acid oxidation (Figures 2(c) and 2(d)).

### 3.3. Construction of the Prognostic Prediction Model Based on the Differential Expressed Genes between Immunity_L and Immunity_H Subtype

**Figure 3:**
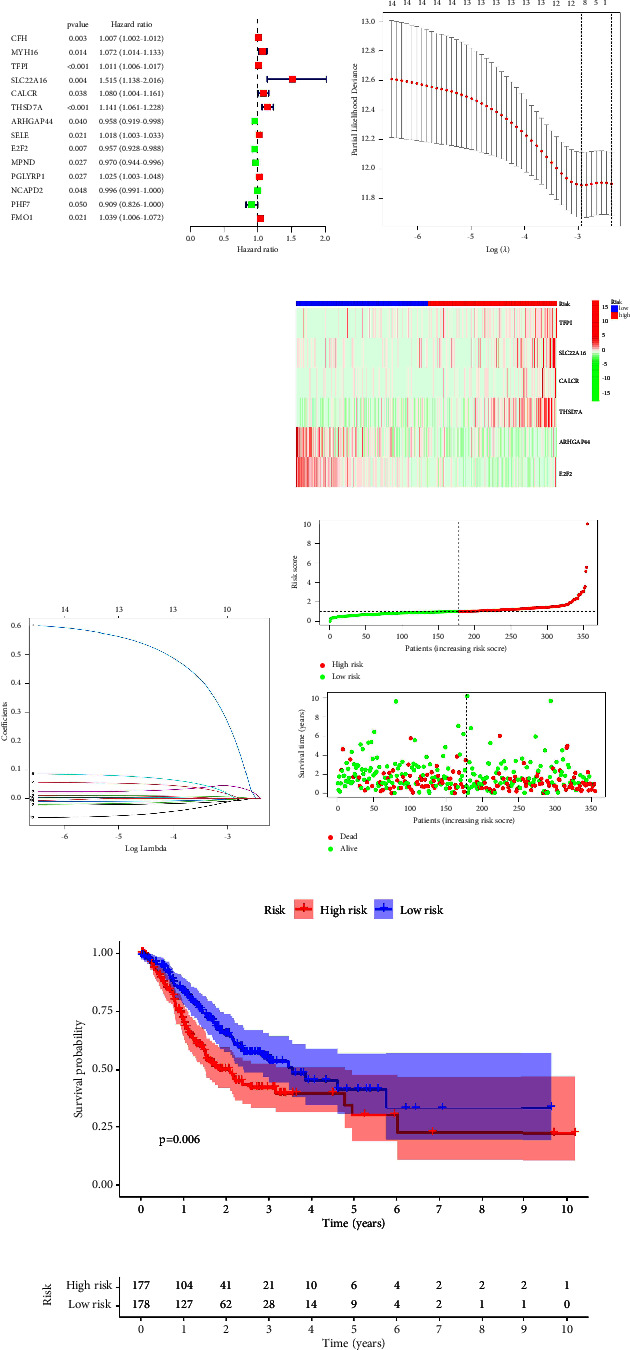
(a) The results of univariate cox regression analysis; ((b)-(c)) the results of lasso regression analysis; (d) the risk plot between low- and high-risk groups; (e) the survival analysis between low- and high-risk groups.

**Figure 4:**
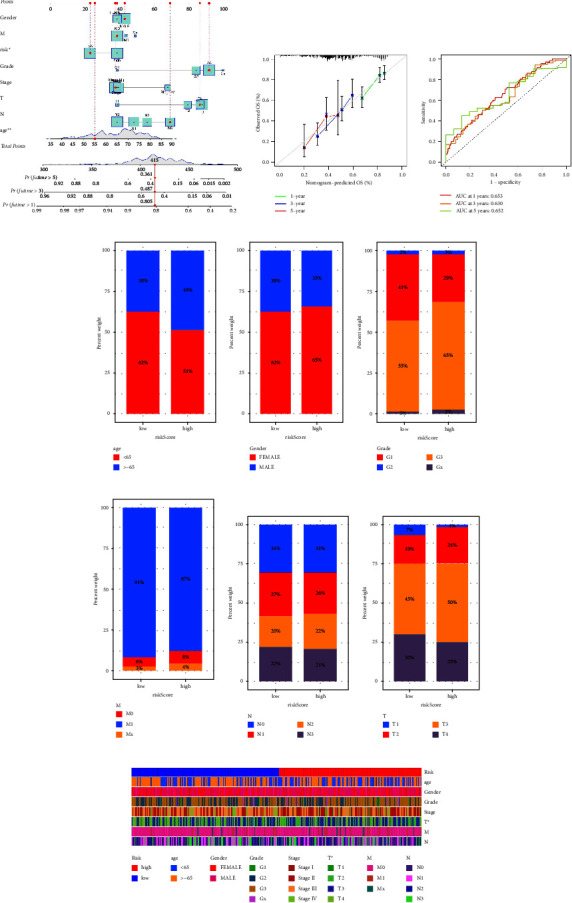
(a) The nomogram was constructed by clinical characteristics and risk score; (b) the calibration curve was applied to reveal the predictive value of nomogram; (c) the time-dependent ROC curve is applied to evaluate the predictive value of prognostic prediction value; (d) the clinical correlation analysis between risk score and age; (e) the clinical correlation analysis between risk score and gender; (f) the clinical correlation analysis between risk score and grade; (g) the clinical correlation analysis between risk score and M stage; (h) the clinical correlation analysis between risk score and N stage; (i) the clinical correlation analysis between risk score and T stage; (j) the heat map demonstrated was applied to show the clinical correlation analysis between risk score and clinical characteristics of GC patients.

The prognostic prediction model is then constructed in order to explore the genes that are closely associated with different immune subtypes. There is a high correlation between 14 genes and the prognosis of patients with GC according to univariate cox regression analysis (Figure 3(a)). In addition, the lasso regression reveals that the model is optimal when lambda is 10, which involves MYH16, TFPI, SLC22A16, CALCR, THSD7A, ARHGAP44, E2F2, MPND, NCAPD2, and PHF7 (Figures 3(b) and 3(c)). Our final step was to perform multivariate Cox regression analysis and to construct a prognostic prediction model based on the risk scores assigned to each patient: risk score = TFPI ∗ 0.0102943363210788 + SLC22A16 ∗ 0.608827192332847 + CALCR ∗ 0.101781283350081 + THSD7A ∗ 0.0710558398763891 + ARHGAP44 ∗ −0.0313871395805361 + E2F2 ∗ −0.0378035205275216. According to the median expression level of risk score, GC patients in the TCGA cohort were divided into low- and high-risk groups (Figure 3(d)). Overall survival (OS) was associated with a lower rate for GC patients in high-risk groups in the survival analysis (Figure 3(e)). For GC patients, the time-dependent ROC curve shows AUC scores of 0.653 (at 1-year), 0.630 (3-year), and 0.652 (5-year), respectively, (Figure 4(c)). In addition, we construct a nomogram based on the age, gender, grade, stage, TMN stage, and risk score of GC patients for better prediction of their prognosis (Figures 4(a) and 4(b)). The correlation analysis was then performed to evaluate the relationship between risk score and clinical characteristics. More than 65-year-old GC patients tend to have lower risk scores (Figure 4(d)). Furthermore, male patients with GC have higher risk scores (Figure 4(e)). GC patients with a lower stage and grade are more likely to join the low-risk group, whereas GC patients with a higher stage do not tend to be members of the low-risk group (Figures 4(e)–4(h)). Also, the results showed that the risk score was closely associated with the T stage (Figure 4(i)). A heatmap shows the relationship between patients' risk scores and clinical characteristics (Figure 4(j)).

### 3.4. Different Immune Scores, Immunotherapy Response between Low- and High-Risk Groups

**Figure 5:**
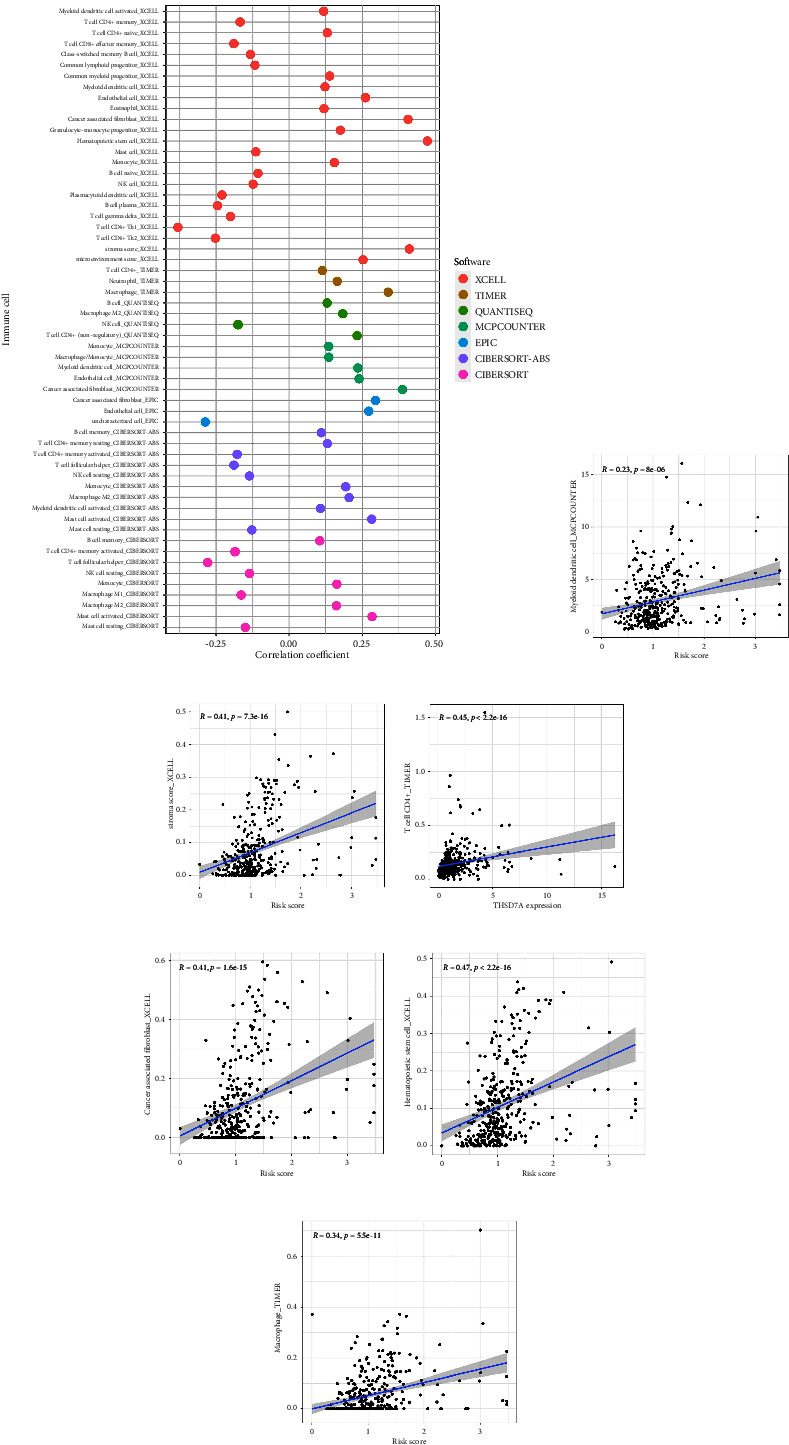
(a) The correlation between multiple immune scores and risk score; (b) the correlation analysis between DC and risk score; (c) the correlation analysis between stromal score and risk score; (d) the correlation analysis between CD4^+^ T cells and risk score; (e) the correlation analysis between cancer-associated fibroblasts and risk score; (f) the correlation analysis between hematopoietic stem cell and risk score; (g) the correlation analysis between macrophages and risk score.

**Figure 6:**
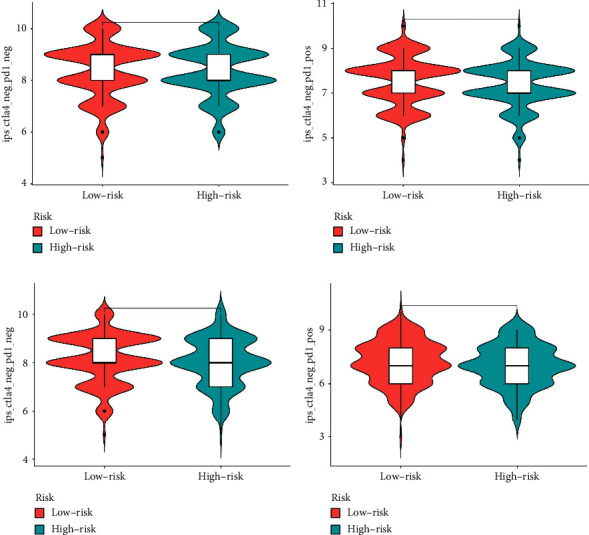
(a) Immunotherapy evaluation of CTLA4 negative and PD1 negative patients between low- and high-risk groups. (b) Immunotherapy evaluation of CTLA4 negative and PD1 positive patients between low-and high-risk groups. (c) Immunotherapy evaluation of CTLA4 positive and PD1 negative patients between low- and high-risk groups. (d) Immunotherapy evaluation of CTLA4 positive and PD1 positive patients between low-and high-risk groups.

**Figure 7:**
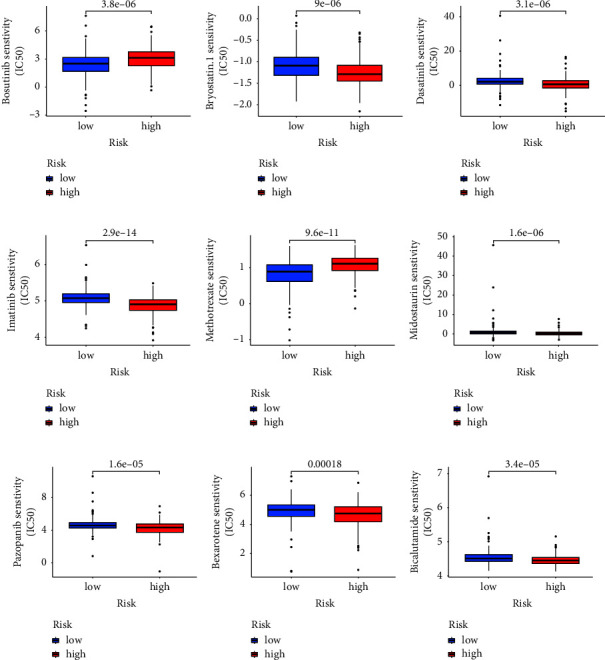
The chemotherapy sensitivity of bosutinib (a), bryostatin.1 (b), dasatinib (c), imatinib (d), methotrexate (e), midostaurin (f), pazopanib (g), bexarotene (h), and bicalutamide (i).

Our next step was to determine the difference between the immune scores of low and high-risk groups. Researchers demonstrated that cancer-associated fibroblasts, hematopoietic stem cells, endothelial cells, monocytes, stromal score, macrophage, mast cell, and microenvironment score are closely related to the risk score (Figures 5(a)–5(e)). There is no significant difference between GC patients with low-risk scores and those with high-risk scores when it comes to immunotherapy response with CTLA4 and PD1 (Figures 6(a)–6(d)). In addition, the R package “pRRophetic” was used to predict the chemosensitivity of GC samples. The results revealed that the prognosis-related prediction model was closely associated with the sensitivity of many drugs, including bosutinib, bryostatin, dasatinib, imatinib, methotrexate, midostaurin, pazopanib, bexarotene, and bicalutamide (Figures 7(a)–7(i)).

### 3.5. Exploration of the Role of E2F2 in the GC Cohort

**Figure 8:**
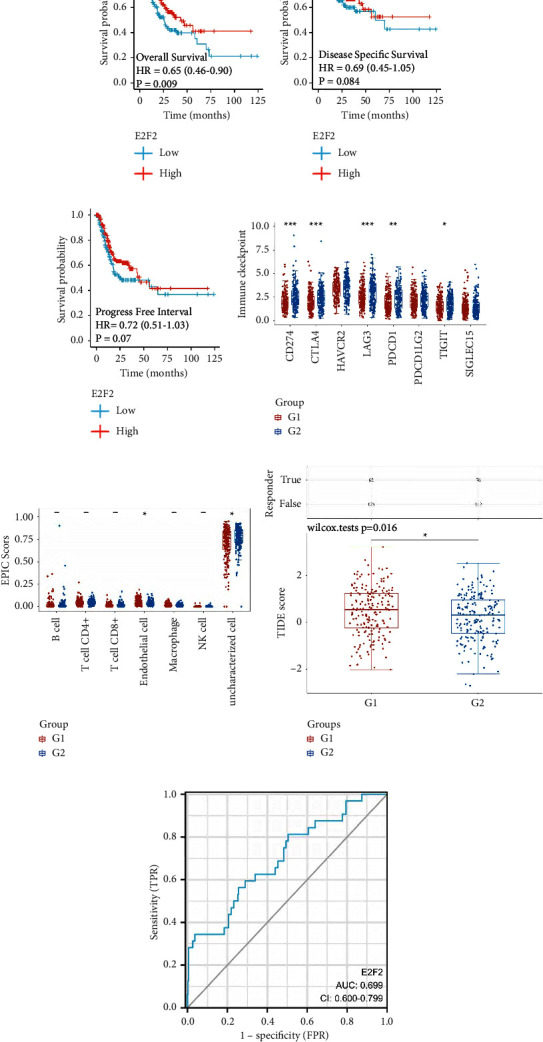
(a) The OS between high- and low-expression of E2F2 groups; (b) the DSS between high- and low-expression of E2F2 groups; (c) the PFI between high- and low-expression of E2F2 groups; (d) the immune checkpoint-related genes between high- and low-expression of E2F2 groups; (e) the immune cell infiltration between high- and low-expression of E2F2 groups; (f) the TIDE score between high- and low-expression of E2F2 groups; (g) the ROC curve shows the predictive value of E2F2 in TCGA GC cohort.

The next step was to determine if there was a correlation between E2F2 expression and GC cohort composition. A high expression of E2F2 is associated with a shorter overall survival (OS) rate and disease-specific survival (DSS) as well as a shorter progression-free interval (PFI) during the survival analysis (Figures 8(a)–8(c)). The immune checkpoint analysis demonstrated that the expression level of E2F2 is associated with CD274, CTLA4, LAG3, PDCD1, and TIGIT, suggesting that E2F2 could be considered as an immunotherapy target for immune checkpoint blocking (Figure 8(d)). According to the immune cell analysis, the expression of E2F2 is associated with endothelial cells (Figure 8(e)). The TIDE score of GC patients with lower levels of expression of E2F2 is associated with lower immunotherapy responses, indicating that GC patients with lower levels of expression of E2F2 may respond less well to immunotherapy (Figure 8(f)). Time-dependent ROC curves showed that E2F2 has good predictive value for GC cohorts (Figure 8(g)).

### 3.6. Functional Analysis Based on the Risk Score and Key Genes

**Figure 9:**
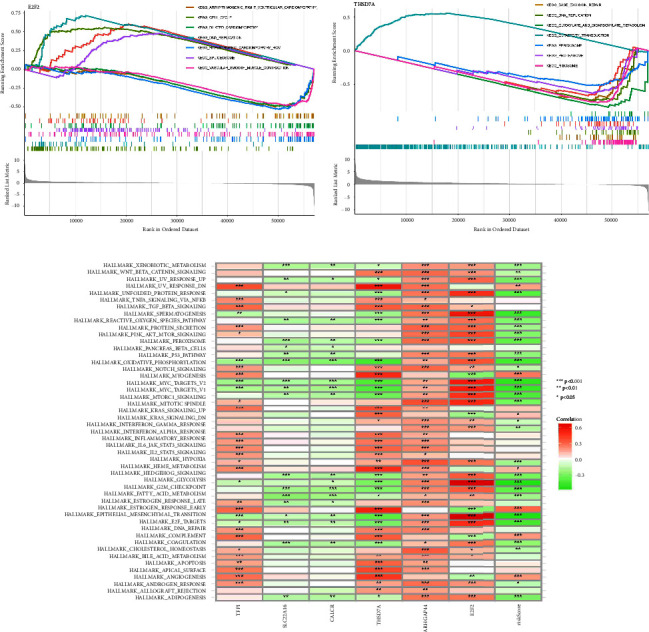
(a) The GSEA analysis based on E2F2; (b) the GSEA analysis based on THSD7A; (c) the GSVA analysis based on the risk score and key genes involved in GC cohort.

After performing GSEA and GSVA enrichment analyses, we looked for potential pathways that are closely associated with the risk score and genes that contribute to prognostic prediction models. The GSEA enrichment analysis demonstrated that E2F2 is closely associated with arrhythmogenic right ventricular cardiomyopathy, cell circle, dilated cardiomyopathy, DNA replication, hypertrophic cardiomyopathy hcm, and spliceosome (Figure 9(a)). For THSD7A, the GSEA enrichment analysis demonstrated that base excision repair, DNA replication, glyoxylate and dicarboxylate metabolism, steroid biosynthesis, calcium signaling pathway, and neuroactive ligand-receptor interaction (Figure 9(b)). Finally, we performed GSVA analysis. The results demonstrated that risk score is associated with many enriched pathways, including adipogenesis, androgen response, angiogenesis, bile acid metabolism, cholesterol homeostasis, coagulation, DNA repair, and E2F target (Figure 9(c)).

## 4. Discussion

It is estimated that there are approximately 1,089,103 new cases of gastric cancer diagnosed each year, making it the sixth most common cancer among newly diagnosed patients [13]. Approximately 133,100 new cases of gastric cancer are diagnosed in Europe each year, and approximately 102,200 deaths are caused by gastric cancer, ranking it fifth among European men and sixth among European women [14]. The survival rate for GC patients is 32% at present. The disease is typically diagnosed after it has spread to other parts of the body, so effective treatment requires a multimodal approach tailored to each individual [15]. Established immunotherapies have been enhanced by recent developments in immune checkpoint inhibition. Several European and American authorities have approved single-agent and combination therapies with PD-1 inhibitors for advanced gastric cancer treatment, whether it is a first- or third-line treatment [16]. For this reason, we must explore potential therapeutic targets to reduce mortality in GC patients. GC cohorts were divided into low- and high-immune groups to explore potential therapeutic targets for immunotherapy. As a result, we evaluated the immune cell infiltration between different subtypes, revealing that Immunity_L subtypes show lower immunity purity. Moreover, the immunological score, estimate score, and stromal scores are all higher with the Immunity_H subtype. For HLA-related genes, the expression levels of most immune HLA genes were significantly reduced in Immunity_L, suggesting that impaired antigen presentation by tumor cells is an evasion mechanism of immune surveillance. A great deal of research has been conducted on the HLA genes in GC patients over the past few years. Several clinical and pathological factors associated with gastric cancer are affected by two antitumor immune markers: Treg infiltration and HLA class I expression. Immunologically, combining HLA class I expression with Treg cell infiltration can provide an improved prediction of postoperative outcomes. In addition, clinical factors associated with gastric cancer are independently influenced by HLA-E and HLA-F. Invasive depth, lymph node involvement, lymphatic invasion, and venous invasion were2 significantly correlated with HLA-E and HLA-F expression.

An analysis of univariate cox regressions, lasso regressions, and multivariate cox regressions was performed to uncover the genes implicated in the genetic composition of GC cohorts. To conclude, a six-signature prognostic prediction model has been developed, which includes TFPI, SLC22A16, CALCR, THSD7A, ARHGAP44, and E2F2. According to the survival analysis, the model is closely associated with GC prognosis. In addition, time-dependent ROC curves demonstrated that the model had good predictive value for GC patients. As a result of many studies in recent years, there have been many discoveries of potential biomarkers for immunotherapy of GC patients that have enabled the construction of the best model for prognosis prediction. There was a decrease in miR-21 and miR-181b expression among patients treated with S-1 and docefluridine [17]. In addition, patients with low miR-125a-3p expression have larger tumors, invade, metastasize, and are at an advanced stage of the disease [18]. To validate the predictive value of our prognostic model, we performed survival analysis, ROC curve analysis, and nomogram analysis, which may provide new sight into the early diagnosis and early treatment of GC patients.

E2F2 has been extensively researched in the past few decades in association with gastric cancer. It has been shown that high E2F2 levels are associated with a poor prognosis [19]. A further function of E2F2 is that it regulates PI3K/Akt/mTOR, which are necessary for cell migration, invasion, and autophagy. According to another study, miR-26a increases GC cell sensitivity to cisplatin-based chemotherapy by targeting E2F2 [20]. This study aimed to determine whether E2F2 is closely associated with the prognosis of GC patients. Next, we explored the pathways that are closely associated with E2F2. DNA replication may be associated with E2F2, according to research, which shows that MTA2 impairs DNA replication stress in gastric cancer cells and increases their sensitivity to PARP inhibition [21].

Bioinformatics analysis has expanded its use in predicting treatment response to gastric cancer, as mRNA may now serve both diagnostic and prognostic purposes. As opposed to a single miRNA signature, multiple-mRNA signatures provide clinicians with valuable information on how to manage the disease in a personalized manner [22]. Our better understanding of these mRNAs and their target genes will allow us to develop more complex and effective therapeutics for gastric cancer. However, the bioinformatics analysis has some inevitable shortcomings. First, we did not provide the verification assays. In addition, no extra dataset was involved in this analysis, which may lead to the heterogeneity of the bioinformatics analysis. Therefore, further analysis should focus on the role of E2F2 in the GC cells. Our analysis provided a new direction for the future exploration of the biomarkers of GC.

## Data Availability

The datasets used and/or analyzed during the current study are available from the corresponding author upon reasonable request.
